# Pulmonary Electrical Injury: A Case Report on an Uncommon Cause of Refractory Hypoxemia

**DOI:** 10.7759/cureus.99435

**Published:** 2025-12-17

**Authors:** Santiago Rivera Castrillón, Jose F Zuluaga, Maria C Florian Perez, Daniel Filizzola, Manuela Orozco

**Affiliations:** 1 Internal Medicine, Universidad de Manizales, Manizales, COL; 2 Cardiology: Internal Medicine, Echocardiography, Advanced Cardiac Imaging, Hospital Santa Sofía, SES (Servicios Especiales de Salud) Hospital Universitario de Caldas, Universidad de Manizales, Manizales, COL; 3 Critical Care Medicine, Internal Medicine, Universidad de Caldas, Manizales, COL

**Keywords:** acute hypoxemia, acute lung injury, critical care, electrical injury, electric injuries, prone position ventilation, pulmonary complications, pulmonary failure, pulmonary hypertension, refractory hypoxemia

## Abstract

Pulmonary involvement after high-voltage electrocution is rare and may present with hypoxemia and pulmonary hypertension out of proportion to imaging, suggesting a vascular/endothelial mechanism. The objective of this report is to describe a case of disproportionate hypoxemia with severe pulmonary hypertension after electrocution, highlight diagnostic pitfalls, and outline management implications.

A previously healthy 46-year-old man sustained high-voltage electrocution, required prolonged resuscitation, and developed severe hypoxemia (PaO₂/FiO₂ (ratio of arterial oxygen partial pressure to fractional inspired oxygen): 68) despite protective ventilation and proning. CT showed only mild posterior ground-glass change; echocardiography revealed preserved right ventricular function. On ICU day 4, a CT pulmonary angiogram (CTPA) identified very distal subsegmental pulmonary embolism. Pulmonary artery catheterization documented pulmonary hypertension (pulmonary artery pressure (PAP): 68/39 mmHg; mean ≈49 mmHg), high/normal cardiac index (4.19 L/min/m²), and mildly elevated pulmonary vascular resistance.

Despite prone positioning (FiO₂ 1.0, positive end expiratory pressure (PEEP) 8 cm H₂O, tidal volume (Vt) ~6 mL/kg predicted body weight (PBW)) and neuromuscular blockade, hypoxemia persisted. Inhaled vasodilators and veno-venous extracorporeal membrane oxygenation (VV-ECMO) were not available at our center. The patient progressed to multiorgan failure and died.

Electrocution may precipitate a primary pulmonary vascular phenotype with physiology that outpaces imaging. Early hemodynamic assessment can refine diagnosis and escalation. Where available, time-limited trials of inhaled vasodilators and timely consideration of VV-ECMO are reasonable.

## Introduction

Electrical injuries, particularly high-voltage exposures, are uncommon but carry substantial multisystem morbidity (cardiovascular, musculoskeletal, and integumentary) [1,2]. Pulmonary involvement is rare and likely underrecognized [1,3,4]. Reported phenotypes span diffuse alveolar hemorrhage and non-cardiogenic pulmonary edema to acute pulmonary hypertension and severe hypoxemia with minimal imaging abnormalities, a pattern consistent with a vascular/endothelial mechanism driven by electroporation-related endothelial injury, acute vasoconstriction, and microthrombosis [1-3,5-7]. Clinically, this may present with profound hypoxemia, elevated pulmonary artery pressures, and preserved right-ventricular function, with CT showing only mild parenchymal change.

Here, we report a high-voltage electrocution followed by disproportionate hypoxemia and marked pulmonary hypertension despite minimal imaging findings and preserved right ventricular (RV) function. We highlight diagnostic pitfalls, including the limited explanatory value of isolated very distal subsegmental PE, and discuss management implications in the ICU (hemodynamic profiling, consideration of inhaled vasodilators, and criteria for veno-venous extracorporeal membrane oxygenation (VV-ECMO)) [1,4-10].

Rationale/novelty

Pulmonary electrical injury with hypoxemia and pulmonary hypertension out of proportion to imaging is under-recognized and diagnostically challenging. By pairing this phenotype with quantitative invasive hemodynamics and a pragmatic escalation pathway, we aim to help clinicians identify and manage similar presentations.

## Case presentation

A 46-year-old male with no relevant medical history sustained a high-voltage electrical injury after direct contact with a primary energy distribution line. A family member trained in basic life support found him unresponsive and pulseless, initiating resuscitation that continued en route to a tertiary care hospital. Upon arrival, he remained in ventricular fibrillation, requiring three 200-joule biphasic defibrillation shocks. Return of spontaneous circulation (ROSC) was achieved after 25 minutes.

Post-resuscitation examination revealed burn marks on the right hand (entry point), occipital scalp, right chest, scapular region, abdomen, and right leg (exit point). In the ICU on admission (day 0), the findings were: blood pressure 109/61 mmHg (mean arterial pressure (MAP) ≈ 77 mmHg), heart rate 123 bpm, oxygen saturation (SpO₂) 93% on invasive ventilation, and temperature 36.5 °C. He remained in shock, requiring vasopressor and ventilatory support.

Initial laboratory findings showed severe mixed acidosis, anuria, markedly elevated creatine phosphokinase (9,214 U/L), and troponin T (27,500 ng/L), as well as active gastrointestinal bleeding (Table 1).

**Table 1:** Initial laboratory investigations with reference ranges PaCO₂: partial pressure of carbon dioxide; AKI: acute kidney injury; CPK: creatine phosphokinase; INR: international normalized ratio

Parameter	Result	Units	Reference range	Comment
Arterial pH	7.23	—	7.35–7.45	Mixed acidosis
PaCO₂	46	mmHg	35–45	—
HCO₃⁻	19.3	mmol/L	22–26	—
Lactate	4.4	mmol/L	0.5–2.0	Elevated
Base excess	−8.3	mmol/L	−2 to +2	—
Urine output (0–24 h)	Anuria	mL/24 h	>0.5 mL/kg/h	Consistent with AKI
Serum creatinine	2.7	mg/dL	0.6–1.3	Consistent with AKI
Urea	25	mg/dL	10–50	—
Potassium	3.5	mmol/L	3.5–5.1	—
Sodium	140	mmol/L	135–145	—
Total CPK	9,214	U/L	20–200	Rhabdomyolysis
Troponin T	27,500	ng/L	<14	Myocardial injury
INR	1.2	—	0.8–1.2	__
Platelets	250	×10⁹/L	150–400	—

**Figure 1:**
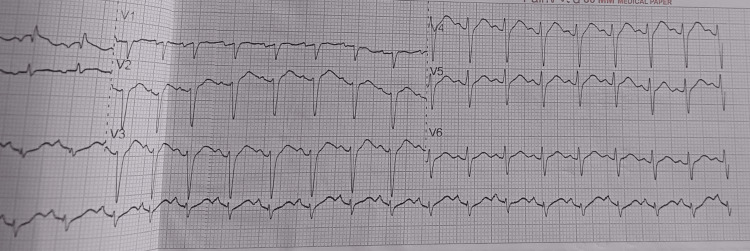
Electrocardiogram on ICU day 1 showing sinus tachycardia (~150 bpm) without conduction or repolarization abnormalities with no ST-segment elevation or depression QTc ~ 352 ms. Paper speed 25 mm/s, gain 10 mm/mV. Digital markers: arrows indicate regular P waves; calibration markers shown. ECG: electrocardiogram; QTc: corrected QT interval

**Figure 2:**
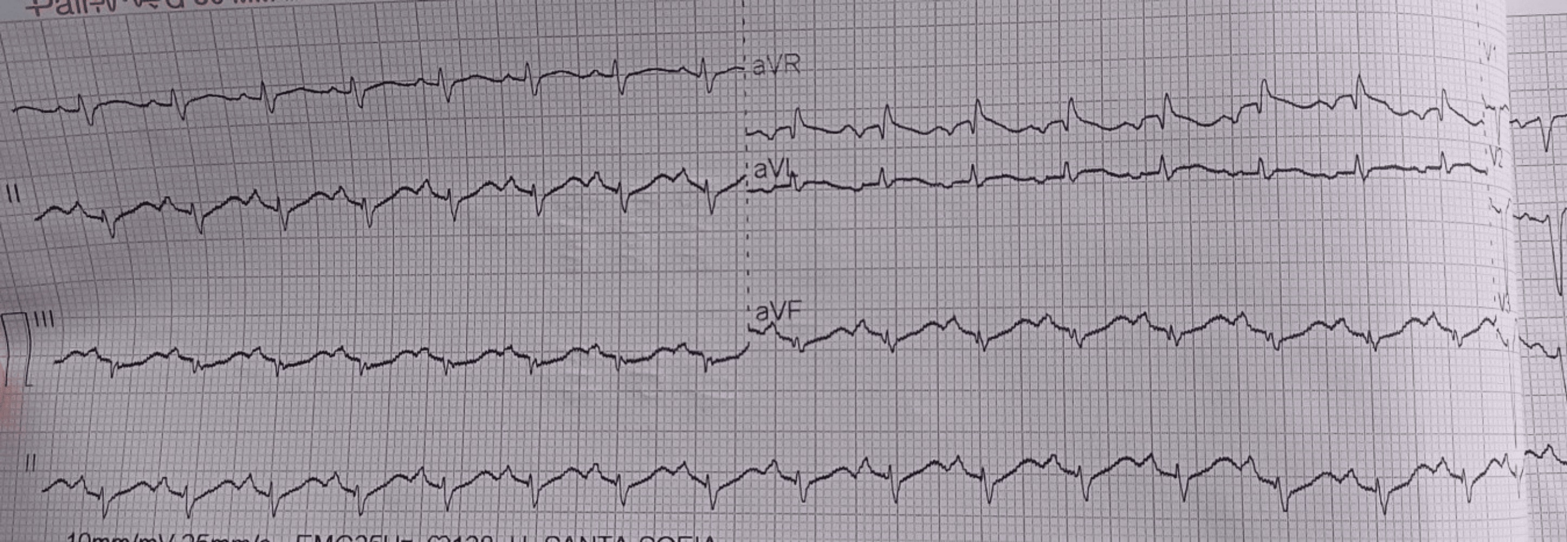
Electrocardiogram on ICU day 1 showing sinus tachycardia (~150 bpm) without conduction or repolarization abnormalities with no ST-segment elevation or depression QTc ~ 352 ms. Paper speed 25 mm/s, gain 10 mm/mV. Digital markers: arrows indicate regular P waves; calibration markers shown. ECG: electrocardiogram; QTc: corrected QT interval

ECG showed sinus tachycardia without conduction or repolarization abnormalities (Figures 1, 2).

**Figure 3:**
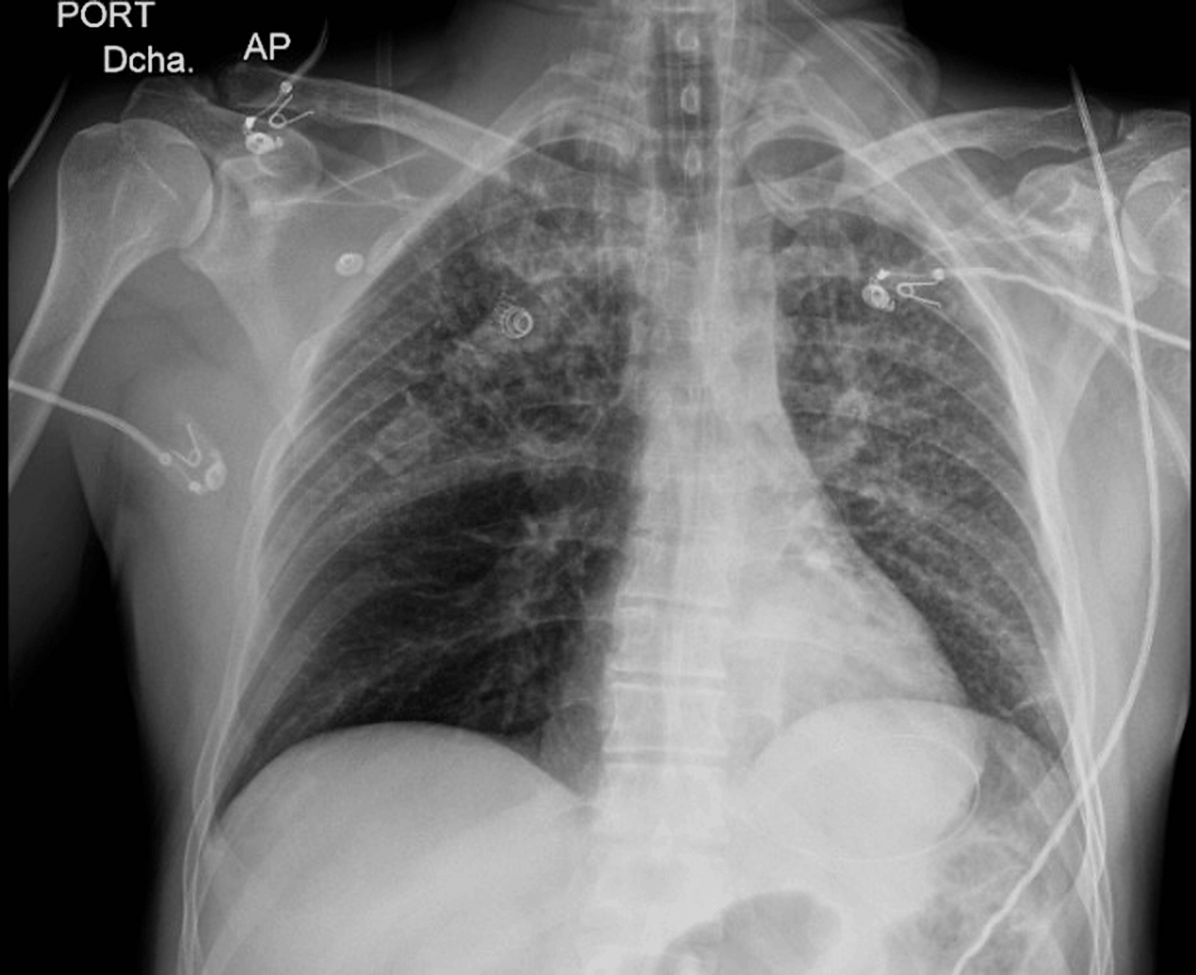
Chest X-ray on ICU day 1 showing mild left-sided alveolar infiltrates

Chest X-ray with mild-moderate alveolar infiltrates (Figure 3). At day 1, a transthoracic echocardiogram demonstrated a hyperdynamic left ventricle with preserved ejection fraction (65%) and normal right ventricular function.

**Figure 4:**
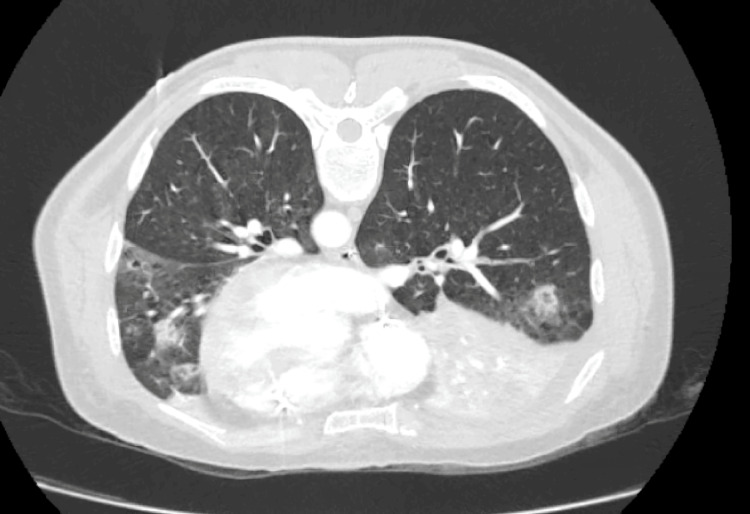
CT pulmonary angiography (axial, lung window) on ICU day 4 Mild posterior ground-glass opacities (boxes) with otherwise preserved aeration is seen. No proximal pulmonary arterial filling defects seen on this slice. Digital markers: boxes highlight ground-glass areas. CT = computed tomography; CTPA = CT pulmonary angiography

**Figure 5:**
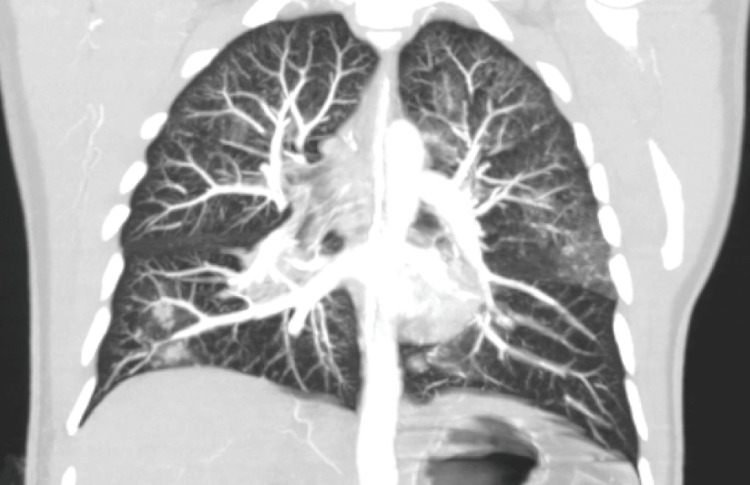
CT pulmonary angiography (coronal MIP, 10-mm slab) on ICU day 4 Very distal SSPE (arrow) seen along a peripheral branch; central and lobar arteries are patent. Coronal MIP enhances visualization of small, contrast-opacified vessels. MIP: maximum intensity projection; SSPE: subsegmental pulmonary embolism

On ICU day 1, PaO₂/FiO₂ declined further, prompting initiation of prone positioning and neuromuscular blockade (cisatracurium). Despite lung-protective ventilation with FiO₂ 1.0, PEEP 8 cm H₂O, and tidal volume ~6 mL/kg PBW, the patient remained severely hypoxemic (PaO₂/FiO₂ 68). CT pulmonary angiography (Figures 4, 5) on day 4 revealed very distal subsegmental pulmonary embolism (SSPE) and mild posterior ground-glass opacities. These findings did not fully explain the respiratory compromise. Pulmonary hypertension was determined by pulmonary artery catheterization: systolic/diastolic PAP 68/39 mmHg, yielding a mean pulmonary arterial pressure (mPAP) of ≈49 mmHg ($\frac{sPAP + 2 \times dPAP}{3}$). Pulmonary capillary wedge pressure (PCWP) was 18 mmHg. With a cardiac index of 4.19 L/min/m², the transpulmonary gradient (TPG) was ≈31 mmHg (mPAP − PCWP), and the diastolic pressure gradient (DPG) was ≈21 mmHg (dPAP − PCWP). Pulmonary vascular resistance (PVR) was ~6.2 Wood units (~495 dyn·s·cm⁻⁵). These values indicate elevated left-sided filling pressure with a substantial pre-capillary component (elevated TPG/DPG and PVR), consistent with combined post- and pre-capillary pulmonary hypertension in the acute setting. We therefore use “pulmonary hypertension” descriptively while emphasizing the prominent pulmonary vascular component.

The patient progressed to multiorgan failure, including hepatic, renal, hematologic, and respiratory involvement. Coagulopathy, anemia, and thrombocytopenia worsened rapidly, culminating in death.

## Discussion

This patient’s course after high-voltage electrocution is best explained by a vascular-dominant acute lung injury, where physiology outpaced imaging. Within hours of ROSC, PaO₂/FiO₂ fell to 68 despite protective ventilation and proning, while CT showed only mild posterior ground-glass change, and transthoracic echocardiography showed a preserved right ventricle. This discordance-profound hypoxemia with minimal parenchymal burden aligns with reports of primary endothelial injury in electrical trauma [1-3,7], and with broader forensic/clinical observations on electrocution pathophysiology [11,12]. Endothelial disruption can drive acute pulmonary vasoconstriction and loss of hypoxic vasoregulation, causing severe V/Q maldistribution (functional shunt) despite relatively intact alveoli [1-3,5,7,11]. A concurrent pro-coagulant milieu likely adds microthrombosis below routine CT resolution, coherently explaining the hemodynamics observed (PAP 68/39 mmHg, CI 4.19 L/min/m²) and the severity of gas-exchange failure.

The very distal SSPE detected on day 4 is unlikely to be the primary cause of hypoxemia. Contemporary guidance notes its limited hemodynamic impact in isolation and the need to balance anticoagulation against bleeding risk and competing diagnoses [9,10]. Here, the preserved RV function and scant parenchymal findings favor diffuse vascular dysfunction rather than macro-embolic physiology.

Management was framed by this vascular lens. Protective ventilation and prolonged proning remained foundational [4,5]. Where pulmonary hypertension and endothelial features predominate, a time-limited trial of inhaled vasodilators (nitric oxide/prostacyclins) is reasonable, with response tracked by PaO₂/FiO₂ and hemodynamics [6,7]. VV-ECMO should be considered when PaO2/FiO2 ≤100 persists despite optimization, per the Extracorporeal Life Support Organization (ELSO) principles and local capacity [8], consistent with acute respiratory distress syndrome (ARDS) escalation frameworks for refractory hypoxemia [13]. In our center, inhaled vasodilators and VV-ECMO were not available, which precluded their use despite physiologic rationale.

This report adds quantitative invasive hemodynamics to an under-recognized presentation--disproportionate hypoxemia with severe pulmonary hypertension and minimal imaging changes--supporting a primary pulmonary vascular injury after electrocution. Limitations include the absence of bronchoscopy with bronchoalveolar lavage (BAL), incomplete serial ventilatory/gas-exchange data, and the local unavailability of inhaled vasodilators and VV-ECMO. Even so, the synthesis of clinical, imaging, and hemodynamic signals provides a coherent, actionable narrative aligned with emerging evidence [1-3,4-10,13]. Our case differs from prior reports by demonstrating severe hypoxemia with minimal parenchymal change and no structural cardiac involvement, absence of explanatory macrothrombosis on CTPA, and detailed invasive hemodynamics (PAP 68/39 mmHg with a high/normal cardiac index) supporting a primary pulmonary vascular injury. The persistent sinus tachycardia despite correction of the usual triggers further underscores ongoing vascular stress rather than parenchymal flooding.

Clinical implications

The clinical implications of this study are: early hemodynamic profiling matters; don’t over-attribute refractory hypoxemia to distal SSPE; consider inhaled vasodilators as a diagnostic-therapeutic probe; and maintain a low threshold for ECMO triage in centers with capability.

## Conclusions

High-voltage electrocution may present as a primary pulmonary vascular injury with hypoxemia and pulmonary hypertension out of proportion to imaging. Recognizing this phenotype has direct management implications--prompt hemodynamic assessment, avoidance of over-attribution to isolated distal SSPE, and consideration of time-limited trials of inhaled vasodilators with early VV-ECMO triage when hypoxemia remains refractory.

Beyond a single case, these observations encourage a pragmatic escalation pathway for similar presentations: (1) confirm severity with serial gas exchange and lung-protective ventilation; (2) evaluate for a vascular pattern using echocardiography and, when available, pulmonary artery catheterization; (3) where pulmonary hypertension predominates with minimal imaging burden, test nitric oxide or inhaled prostacyclins for physiologic response; and (4) if PaO2/FiO2 remains ≤100 or work of breathing is unsustainable, assess the transfer or initiation of VV-ECMO according to institutional capability and contemporary standards. Future studies should better define the incidence of pulmonary vascular phenotypes after electrical injury, elucidate biomarkers of endothelial damage, and determine which patients derive the greatest benefit from vasodilator therapy or extracorporeal support.
